# A CW-CNN regression model-based real-time system for virtual hand control

**DOI:** 10.3389/fnbot.2022.1072365

**Published:** 2022-12-21

**Authors:** Zixuan Qin, Zixun He, Yuanhao Li, Supat Saetia, Yasuharu Koike

**Affiliations:** ^1^Department of Information and Communications Engineering, Tokyo Institute of Technology, Yokohama, Japan; ^2^Institute of Innovative Research, Tokyo Institute of Technology, Yokohama, Japan

**Keywords:** channel-wise CNN (CW-CNN), real-time control system, regression model, surface electromyography (SEMG), target achievement control (TAC), virtual hand control

## Abstract

For upper limb amputees, wearing a myoelectric prosthetic hand is the only way for them to continue normal life. Even until now, the proposal of a high-precision and natural performance real-time control system based on surface electromyography (sEMG) signals is still challenging. Researchers have proposed many strategies for motion classification or regression prediction tasks based on sEMG signals. However, most of them have been limited to offline analysis only. There are even few papers on real-time control based on deep learning models, almost all of which are about motion classification. Rare studies tried to use deep learning-based regression models in real-time control systems for multi-joint angle estimation *via* sEMG signals. This paper proposed a CW-CNN regression model-based real-time control system for virtual hand control. We designed an Adaptive Kalman Filter to smooth the joint angles output before sending them as control commands to control a virtual hand. Eight healthy participants were invited, and three sessions experiments were conducted on two different days for all of them. During the real-time experiment, we analyzed the joint angles estimation accuracy and computational latency. Moreover, target achievement control (TAC) test was applied to emphasize motion regression in real-time. The experimental results show that the proposed control system has high precision for 3-DOFs motion regression in simultaneously, and the system remains stable and low computational latency. In the future, the proposed real-time control system can be applied to actual prosthetic hand.

## 1. Introduction

Upper limb amputations can be very cruel to a person and can cause inconvenience to life and work. In the United States, approximately 1 of every 200 people living with a limb loss; and according to a survey conducted in 2011, in Japan, approximately 0.0047% of the disabled population in Japan are upper limb amputees (Kato et al., 2016; Frontera et al., 2019). After receiving treatment, it is critical that upper limb amputees actively use prosthetic hands in order to return to work. According to a detailed postal questionnaire which asked amputees about the frequency and satisfaction of wearing functional prostheses, 64% of them rated their prosthetic devices as “fair” or “not acceptable,” and 56% of them wore their limbs “once in a while” or “never” (Davidson, 2002). Upper limb amputees can rely on myoelectric prosthesis controlled by surface electromyography (sEMG) signal. In recent years, sEMG-based top-level commercial prostheses have been released, such as the Michelangelo Hand, Softhand Pro and the open-source 3D hand (Godfrey et al., 2018; Selvan et al., 2021; Bayrak and Bekiroglu, 2022). However, the control methods of such advanced prostheses are rudimentary, the performance is far from natural control and complicated training procedures are required (Atzori et al., 2014). The control systems of the myoelectric prosthetic hand not only limit the quality of amputees' daily life, but also result in the low acceptance. Therefore, it is crucial to investigate a real-time control system with high-precision and natural performance based entirely on sEMG signals.

Many researchers have proposed various real-time control systems for prosthetic hands. For example, Synergy-based (Furui et al., 2019) and Multilayer Perception classifier-based (de Oliveira de Souza et al., 2021) prosthetic hand control systems have been proposed in recent years. Nowadays, many scientists have tried to use deep learning models to decode sEMG signals and to apply these models for motion recognition. However, although there are many recent papers on offline analysis using deep learning methods for motion recognition (Zia ur Rehman et al., 2018; Huang and Chen, 2019; Koch et al., 2020; Bai et al., 2021), few papers discussed the application of those deep learning models in real-time control system. Here are some recent studies on real-time control systems for myoelectric prostheses based on deep learning methods: Jafarzadeh et al. (2019) developed a novel prosthetic hands control system using a convolutional neural network (CNN) motion classifier with 99.98 and 91.26% training and validation accuracy, respectively, however, the test accuracy was low (48.40%); Tam et al. (2020) proposed a CNN-based real-time gesture recognition system for multi-articulating hand prosthesis control with real-time classification accuracy of 98.15% and classification latency of 100–200 ms. However, unfortunately, neither of these papers completed the demonstration results or videos of applying the proposed control system to virtual hands or real prosthetic hands. In addition, we can find very few research results on this topic, almost all of them used CNN for real-time motion classification, and none of them tried to use deep learning-based regression model in real-time control system for multi-joint angles estimation by sEMG signal.

Our previous research (Qin et al., 2021) proposed a channel wise CNN (CW-CNN) regression model for three degrees of freedom (3-DOFs) joint angles estimation, and we not only discussed the high accuracy of offline regression performance, but also proved that the robustness of the proposed model on different days can be maintained and even improved by applying transfer learning. However, even when offline performance looks good, it can exist some significant real-time difference (Simon et al., 2011). Transforming offline analysis into online decoding still remains challenging. Thus, in this work, our goal is to apply the CW-CNN regression model to the proposed real-time control system and evaluate its real-time performance (accuracy and latency) and demonstrate its usability in virtual hand control.

In order to clearly distinguish the proposed method from real-time motion classification methods, we need to demonstrate that the system is able to control the virtual hand to reach some specified target positions and maintain for a period of time in real-time environment. Therefore, it is necessary to consider the method which can track estimated trajectory and emphasize the motions in 3-DOFs simultaneously. Target achivement control (TAC) test has proven to be a useful and challenging method for testing real-time classification performance (Simon et al., 2011; Gusman et al., 2017), however, real-time regression will be more challenging than classification during TAC test. Thus, we applied TAC test on evaluating our real-time control system to emphasize the regression motions.

**Figure 1:**
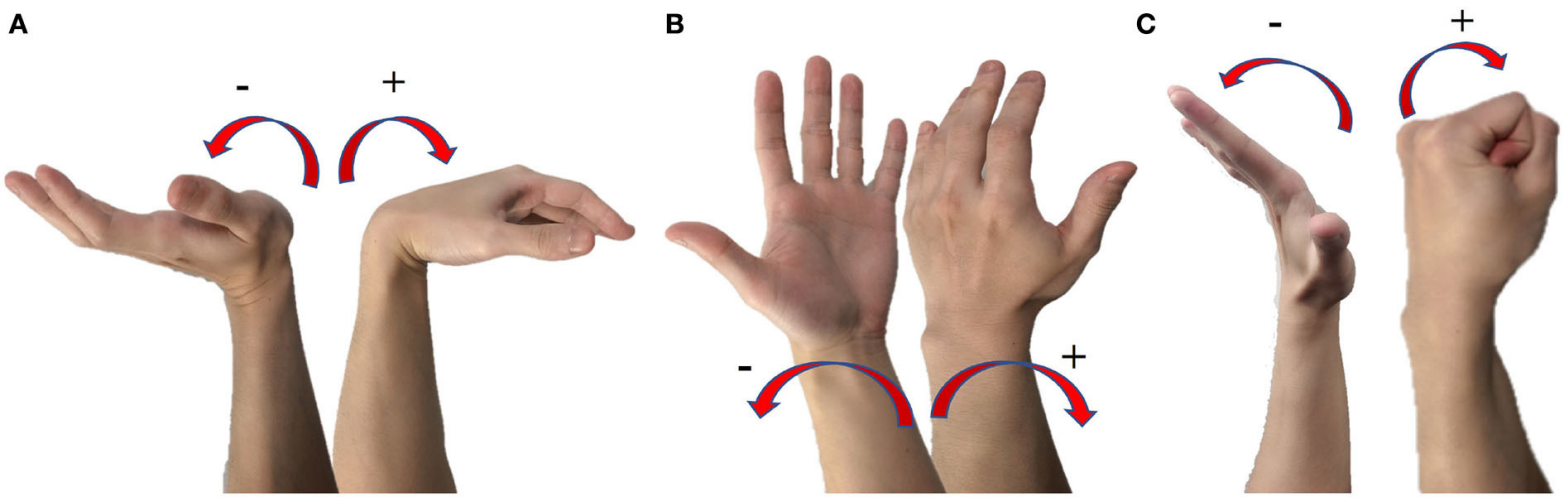
Introduction of the three degrees of freedom used in this study. **(A)** DOF1 is the joint to perform wrist flexion and wrist extension, wrist flexion angle is positive number, and wrist extension angle is negative number. **(B)** DOF2 is the joint to perform wrist pronation and wrist supination, wrist pronation angle is positive number, and wrist supination angle is negative angle. **(C)** DOF3 is the joint to perform wrist grip or return to rest motion, the angle hand grip shows positive number.

In this study, we present a real-time control system for virtual hand control that applies the CW-CNN regression model to estimate 13 daily motions which represented by 3-DOFs (Figure 1) joint angles in real-time. Our goal is to achieve accurate regression prediction not only for single motions but also for mixture motions in real-time, and to control a virtual hand as real-time control demonstration. A sliding window is used to process the sEMG signal received through the lab streaming layer (LSL) in real time. The input of the system is the normalized integrated EMG (IEMG) signal processed from the raw sEMG signals in the sliding window. The estimated joint angles were transmitted to an Adaptive Kalman Filter to reduce oscillations so that prevent motor damage. The smoothed outputs can be sent to a virtual hand as control commands. Our system estimates complex daily motions in real-time, any prosthetic/virtual hand can be used for demonstration. In the real-time experiments, we collected the estimated joint angles, the real joint angles and computational latency of the proposed system *via* LSL, calculated the correlation coefficient, and analyzed the stability of the computational latency of the system using one-way analysis of variance (ANOVA). We evaluated the reliability and validity of the proposed real-time control system in eight healthy participants by conducting three sessions of experiment on two different days. In addition, we designed TAC test to investigate the real-time regression effect of the control system, and the experimental results demonstrated the good performance in real-time. In the future, the proposed real-time control system can be applied to prosthetic hand.

## 2. Materials and methods

### 2.1. Data acquisition devices

The two data acquisition devices are the same as previous work (Qin et al., 2021), or readers can refer to Supplementary Figure S1 for details. The bipolar multi-array electrode (SMK Corp., SEIREN Co., Ltd.; Supplementary Figure S1A) was used to collect raw sEMG signals, the sampling rate of sEMG acquisition was set to 500 Hz due to limitation of signal transmission speed of Bluetooth low energy. The Perception Neuron Motion Capture System (Noitom Ltd., China; Supplementary Figure S1B) was used to measure joint angles in 3-DOFs, and the sampling rate was 120 Hz.

### 2.2. Real-time control system

**Figure 2:**
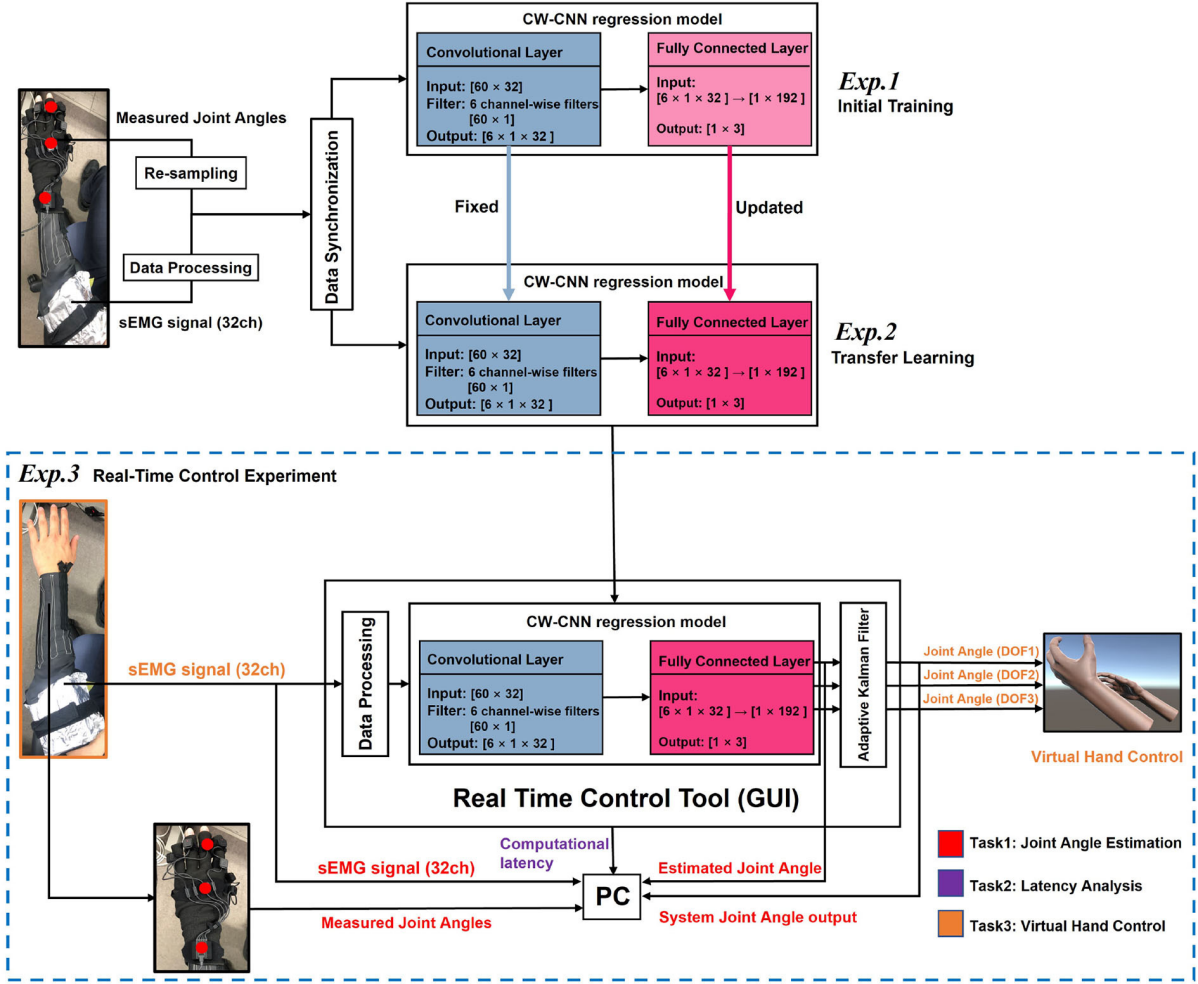
Illustration of the proposed real-time control system. The real-time control consists of three sessions, where ***Exp.1 *** and ***Exp.2 *** show the model training procedures before real-time control (***Exp.3***). The ***Exp.3 *** (part in the blue dashed box) shows the structure of the real-time control system, consists of four parts: Data Processing Module, trained CW-CNN regression model, Adaptive Kalman Filter, and a Virtual Hand model program.

The part of Figure 2 with the blue dashed box shows the composition of the proposed real-time control system. The real-time control tool is a graphical user interface (GUI) that receives sEMG signals in real-time and converts them into the output joint angles of 3-DOFs after internal calculations. The output joint angles can be sent directly to the virtual hand as control commands to control its 3-DOFs respectively. There are three experiments in two different days, we named them as ***Exp.1***, ***Exp.2***, and ***Exp.3***, respectively. Since the results and effects of ***Exp.1 *** and ***Exp.2*** have been discussed in the previous paper (Qin et al., 2021), this paper will only analyze the results and effects of ***Exp.3 *** (blue dotted box), i.e., real-time joint angle estimation and virtual hand control. We will explain the experiment details in the later sections.

The real-time control system was built in a laptop (MouseComputer, CO., LTD, Japan), the operation system was Microsoft Windows 10 Home 64 bit, with NVIDIA GeForce GTX 1660 Ti, the processor was Interl(R) Core(TM) i7-10750H CPU @ 2.60GHz, RAM 32.0 GB. Moreover, the virtual hand was made by Unity Engine (version 2021.3.2f1).

The real-time control GUI tool written in Python3 and PyQt5 (PyPI, Python Software Foundation. https://pypi.org/project/PyQt5/) by our team and a virtual hand model built by Unity (Unity Technologies). The real-time control GUI tool includes Data Processing Module, a regression predictor, and an Adaptive Kalman Filter. The Data Processing Module processes raw sEMG signal in real-time as input to the regression model updated by transfer learning, which will calculate the 3-DOFs joint angles as output. To prevent the oscillation of the estimated joint angles from damaging the motors of prosthetic hand, even though we controlled a virtual hand in this paper, we consider to apply Adaptive Kalman Filter to smooth the joint angles before sending it to the robotics or virtual hand. Please refer to Supplementary Movie S1 for the usage of the proposed real-time control GUI. The following sections will explain each module in detail.

### 2.3. Data processing module

In real time, sEMG signals are processed as normalized integrated EMG (IEMG) signals and used as input to our proposed regression model to estimate the 3-DOFs joint angles. The window size of each sEMG acquisition is 120 ms, i.e., the raw sEMG size of each acquisition is [60 × 32] in each time, 32 is channel numbers, and 60 = 500 Hz × 0.12 s. The real-time control GUI always obtains the latest sEMG signal as input to the Data Processing Module after processing the previous sEMG signal to prevent signal delay.

Before starting the real-time experiment, we need to collect maximum voluntary contraction (MVC) data from the participant for the day for signal normalization afterwards. Firstly, the low-pass finite impulse response (FIR) filter proposed in a previous paper from our laboratory (Koike and Kawato, 1995) is applied to filter the rectified sEMG signals in each channel, which we call them IEMG signals. Then, IEMG signals in each channel are normalized from 0 to 1 *via* the maximum and minimum value of MVC in each channel. This progress can be described as the following equations:


(1) $$\begin{array}{l} IEMG_{i}=filter\left(abs\left(EMG_{i}\right)\right) \end{array}$$

(2) $$\begin{array}{l} IMVC_{i}=filter\left(abs\left(MVC_{i}\right)\right) \end{array}$$

(3) $$\begin{array}{l} IEMG_{norm}^{i}=\frac{IEMG_{i}-min\left(IMVC_{i}\right)}{max\left(IMVC_{i}\right)-min\left(IMVC_{i}\right)} \end{array}$$

where *i* is channel, *i* = 1, 2, ..., 32. *IMVC* is integrated MVC, means to process MVC signals in the same way as IEMG signal, *IEMG*_*norm*_ is the normalized IEMG signal. The *abs*() function means to calculate the absolute value of input series, so that obtain rectified sEMG signals; the *filter*() is the FIR filter which proposed in Koike and Kawato (1995). The size of normalized IEMG data is also [60 × 32], and is fed into the following regression model as input. In real-time experiment, one of the sEMG data processing results is shown in Supplementary Figure S2.

### 2.4. CW-CNN regression model

In our previous research (Qin et al., 2021), we proposed a CNN-based regression model for the estimation of 3-DOFs joint angles, and obtained very high-performance accuracy in offline analysis. We considered the use of channel wise filters in the convolutional layer, which is the reason to name it CW-CNN (Sakhavi et al., 2018).

In this paper, we applied the proposed CW-CNN regression model to our real-time control system to achieve the desired virtual hand control. The model has two layers, a convolutional layer and a fully connected layer. The input to this model is a [60 × 32] matrix, with six [60 × 1] channel wise filters compressing the time dimension to obtain six [1 × 32] feature maps (force pattern). The fully connected layer connects the six force patterns from end to end into a [1 × 192] vector, which is calculated as a 3-DOFs joint angles output by different sets of three parameters (weight and bias) of the fully connected layer, respectively.

In ***Exp.1***, model was initially trained for each participant by 10-fold cross validation on 10-trial datasets. In ***Exp.2***, model of each participant was updated on different days using 5-trial new datasets *via* transfer learning for 5-fold cross validation. After transfer learning, the updated model was applied to the real-time control GUI for joint angles estimation directly in ***Exp.3***. The training and application steps of the above model can be reflected according to Figure 2, and the color of each convolutional layer or fully connected layer indicates whether the layer parameters were updated by training. The detail information about model training is shown in Supplementary Table S1. The training procedure was conducted in different PC, using PyTorch 1.3.1, GeForce RTX 2080 GPU and CUDA10.1. The convolutional layer is denoted as *Conv*. layer and the fully connected layer is FC layer. LR means learning rate, and CV is abbreviation of cross-validation.

### 2.5. Adaptive Kalman filter

In engineering, Kalman filter is often used to estimate the unknown variables more precisely using a series of data (Kalman, 1960; Li et al., 2015), which is an iterative process. Each time a new observation is obtained, system state (*x*) and error covariance (*P*) are updated. Since each iteration uses only the result of the previous iteration and the new measurements, the filter occupies very few computational resources. The filtering process of Kalman Filter can be iterated by the following three formulas:


(4) $$\begin{array}{l} K\left(k\right)=\frac{P\left(k-1\right)}{P\left(k-1\right)+R} \end{array}$$

(5) $$\begin{array}{l} x\left(k\right)=x\left(k-1\right)+K\left(k\right)\cdot \left(z\left(k\right)-x\left(k-1\right)\right) \end{array}$$

(6) $$\begin{array}{l} P\left(k\right)=\left(1-K\left(k\right)\right)\cdot \left(P\left(k-1\right)\right)+Q \end{array}$$

Where, at time *k*, *K*(*k*), *x*(*k*), *P*(*k*), and *z*(*k*) are the Kalman gain, the filtered value we expect, the filter deviation matrix, and observation value, respectively. *Q* and *R* are transition covariance matrix and observation covariance matrix, respectively. However, we need to provide *Q* and *R* values by ourselves. It is hard to say that the preset values are the optimal solutions for our proposed system, therefore, we need the Kalman Filter to have adaptive adjustment. To achieve the Adaptive Kalman Filter (Mehra, 1970), *Q* and *R* need to be updated after each iteration, and we designed the adjustment procedure as the following formulas:


(7) $$\begin{array}{l} Q\left(k+1\right)=Q\left(k\right)+\frac{1}{k+1}\left(\hat{Q}\left(k+1\right)-Q\left(k\right)\right) \end{array}$$

(8) $$\begin{array}{l} R\left(k+1\right)=R\left(k\right)+\frac{1}{k+1}\left(\hat{R}\left(k+1\right)-R\left(k\right)\right) \end{array}$$

Where *k*+1 denotes the next iteration time; *Q*(*k*+1) and *R*(*k*+1) denote the updated values that will be used in next iteration. *Q*(*k*) and *R*(*k*) are the parameters used in *k*th iteration; $\hat{Q}\left(k+1\right)$ and $\hat{R}\left(k+1\right)$ are the variances estimates using the previous *Q* and *R*, respectively.

### 2.6. Participants

We invited eight healthy participants (Sub.1–Sub.8) to participate in the experiment on different days. They were asked to read the participant information sheet and provide written informed consent to participate in this study before the experiments. Due to the limited size of the multi-array electrode sleeves, the choice of the right or left hand depends on whether the forearm size of the participant fits better in the left- or right-hand sleeve. Data from the participants were acquired at the Tokyo Institute of Technology, the study protocol was approved by the ethics committee of the Tokyo Institute of Technology and was conducted in accordance with the Declaration of Helsinki. The participants' details can be found in Supplementary Table S2.

### 2.7. Experiment

The experiment in this study was divided into three sessions: two offline sessions (***Exp.1*** and ***Exp.2***) and real-time control session (***Exp.3***). For each participant, the experiments were conducted on two different days. ***Exp.1 *** aimed to collect data set to train a new joint angle estimation model for each participant. We designed ***Exp.2 *** and ***Exp.3 *** for each participant on other days for real-time control of the virtual hand. Before the real-time control, we collected small number of new datasets to update model parameters *via* transfer learning (***Exp.2***). Then, the updated regression model was applied to the real-time control system, so that participants were able to control the virtual hand in real-time (***Exp.3***). Lab streaming layer (LSL, https://github.com/sccn/labstreaminglayer) is a system for the uniform collection of measurement time series in research experiments. In all sessions of the experiment, LSL was used to synchronize data for model training, result analysis, and control command transmission.

**Figure 3:**
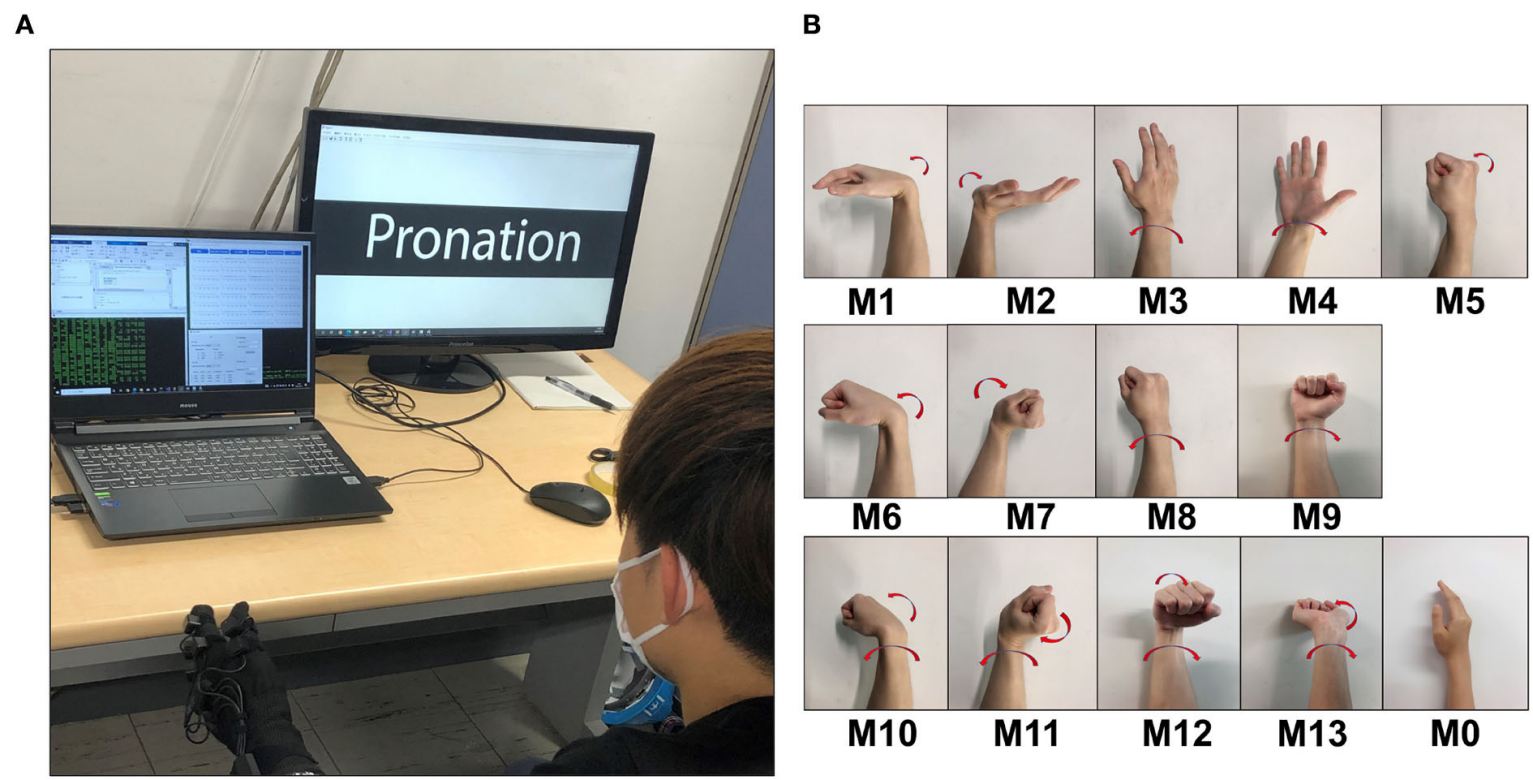
Experiment scene for ***Exp.1 *** and ***Exp.2***. **(A)** A participant (Sub.1) was wearing the electrode sleeve and Perception Neuron glove, seated in front of a screen. The screen will show the next motion need to be performed. **(B)** Daily movements pictures (right hand), show the details of M0 M13 in Table 1. M1–M5 are single motions, M6–M9 are double-mixture motions, and M10–M13 are triple-mixture motions, M0 denotes the central position.

After wearing the devices, the participants sat in front of a screen during the experiment, and they were instructed to perform the corresponding motions displayed on the screen (Figure 3A). The motions were the common daily motions (Figure 3B and Table 1), including five single motions (M1-M5), four double-mixture motions (M6-M9) and four triple-mixture motions (M10-M13), the pictures of each motion (right hand) are given (Figure 3B).

**Table 1:** Specified daily motions that should be performed in the experiment.

**Motion ID**		**Contents**
M0		Rest
Single motions	M1	Wrist flexion (WF)
	M2	Wrist extension (WE)
	M3	Wrist pronation (WP)
	M4	Wrist supination (WS)
	M5	Hand grip (HG)
Double-mixture	M6	WF+HG
	M7	WE+HG
	M8	WP+HG
	M9	WS+HG
Triple-mixture	M10	WF+WP+HG
	M11	WE+WP+HG
	M12	WF+WS+HG
	M13	WE+WS+HG

#### 2.7.1. *Exp.1* and *Exp.2*: model initial training and transfer learning

In ***Exp.1***, there were 10 trials in total. In each trial, participants were required to perform motions from M1 to M13. After each trail, participants rested for approximately 2 min to prevent muscle fatigue.

***Exp.2 *** was conducted in different days, there were 5 trials in total. During the transfer learning, we fixed the convolutional layer parameters and updated the fully connected layer parameters using the new dataset. This transfer learning method is called layer transfer. Layer transfer (Yosinski et al., 2014) is the process of fixing some layers of a model directly into a new network, and then training the rest of the network with only a small amount of new data. In our experiments, we used 32 channels sleeves for sEMG signal acquisition, the sleeve completely encased the entire forearm, and we did not need to adjust the electrode location. Thus, we considered the convolutional layer as the layer in direct contact with the raw sEMG signal, which could acquire the same motion pattern on different days, so we fixed the parameters of convolutional layer and only updated the fully connected layer to maintain the robustness of the model. More discussions and comparison results about the proposed transfer learning methods can be found from Qin et al. (2021). The training of transfer learning process lasted about 2.5 min, so participants did not need to wait too long before proceeding to the real-time estimation experiment. The model training details can be found in Supplementary Table S1.

For both ***Exp.1 *** and ***Exp.2***, after measuring the sEMG signals and joint angles, sEMG signals were processed as normalized IEMG signals, and synchronized with joint angle data *via* time stamps of LSL. However, the sampling rates of sEMG signal and joint angles were different. Therefore, we up-sampled joint angles data from 120 to 500 Hz to match EMG signals. Readers can get more details about ***Exp.1 *** and ***Exp.2 *** in Qin et al. (2021).

#### 2.7.2. *Exp.3*: Real-time experiment

After the transfer learning (***Exp.2***), we obtained an updated regression model for real-time estimation and control. We ran the proposed real-time control system, collected MVC of the participant for signal normalization, loaded the trained model into the system, then we could check the estimated 3-DOFs joint angles in real-time on the GUI tool (Supplementary Movie S1). Then, we connected the real-time system to Unity program, and the system outputs were sent to control the virtual hand in real-time. Participants needed to perform the motions in Table 1. We designed three tasks (Figure 2) of this session: (1) Task 1: Joint angle estimation; (2) Task 2: Computational latency analysis; (3) Task 3: Virtual hand control demonstration;

For Task 1, participants needed to wear Perception Neuron glove, the measured angles, raw sEMG signals, estimated joint angle and the system outputs were acquired *via* LSL for evaluation. Since this was a regression prediction task, we chose Pearson correlation coefficient (CC) (Taylor, 1990; Stapornchaisit et al., 2019; Qin et al., 2021; He et al., 2022) as the metric to evaluate the prediction accuracy:


(9) $$\begin{array}{l} CC\left(X,Y\right)=\frac{1}{N-1}\overset{N}{\underset{i=1}{\sum }}\left(\frac{X_{i}-\mu_{X}}{\sigma_{X}}\right)\left(\frac{Y_{i}-\mu_{Y}}{\sigma_{Y}}\right) \end{array}$$

Where *N* is series length, *X* and *Y* are the variables we need to compare. μ_*X*_ and μ_*Y*_ are mean value of variable *X* and *Y*, respectively; σ_*X*_ and σ_*Y*_ are standard deviation of *X* and *Y*. CC ranges from –1 to 1, with CC = 0 indicating no relationship between the two variables; a positive CC indicates a positive correlation, while a negative CC indicates negative correlation, which is not expected. Therefore, we hope to demonstrate the high regression accuracy of the real-time motion estimation by increasing the CC value to get as close as possible to 1.

We measured the latency of each calculation process in Task 2 (see Section 4.3 and Pseudo Code in Supplementary material), including the processing from sEMG signal to final system output (after Adaptive Kalman Filter). For each participant, the delays were saved as data streaming during the experiment. Participants were instructed to continue doing any motions for 5 min, then computational latency data were saved as a 5-min stream for statistical analysis.

We designed TAC test for analyzing the real-time regression effect, which will be introduced in Section 2.9.

### 2.8. One-Way ANOVA

One-way ANOVA is an appropriate method for more than two groups comparison (Kim, 2014). For each participant, computational latencies were series data in 5 min and divided into five groups, each group representing a sequence of latencies per minute, then we used *anova*1() function in MATLAB (The MathWorks, Inc., USA) to obtain *p*-value and the box plot of data by group. We set the significance level of the ANOVA tests at 0.5 (Ganesh et al., 2018) to evaluate the computational latencies of the five groups and the eight participants: (1) Comparison between minutes: if the system is unstable, the delay also becomes more pronounced over time, this step is to demonstrate the stability of the proposed system during use; (2) Comparison between participants: participants joined the experiments in different days, thus, this step is to demonstrate the stability of the system in different days. If the *p* > 0.5, it can be demonstrated that there is no significant difference between the compared items, namely, the computational latency of the proposed real-time control system was stable, did not vary with time.

### 2.9. TAC test

It is important to provide more proofs of real-time motion regression in this study. Simon et al. (2011) proposed the TAC test to simulate the possible myoelectric prosthetic hand performance in real life. During the TAC test, the participants needed to control the virtual hand in multi-DOFs to touch the target postures, and stayed for a period of time (dwell time). Participants might experience some unexpected regression control feedback, and needed more time to adjust the posture, and thus increased the TAC completion times and decreased the completion rates. According to Simon et al. (2011), we should pay more attention on parameters such as target width (acceptance tolerance), dwell time and trial time limits. Moreover, we used completion rates, completion time and real-time trajectory of the virtual hand as evaluation metrics to evaluate the effect of the proposed regression control system.

**Figure 4:**
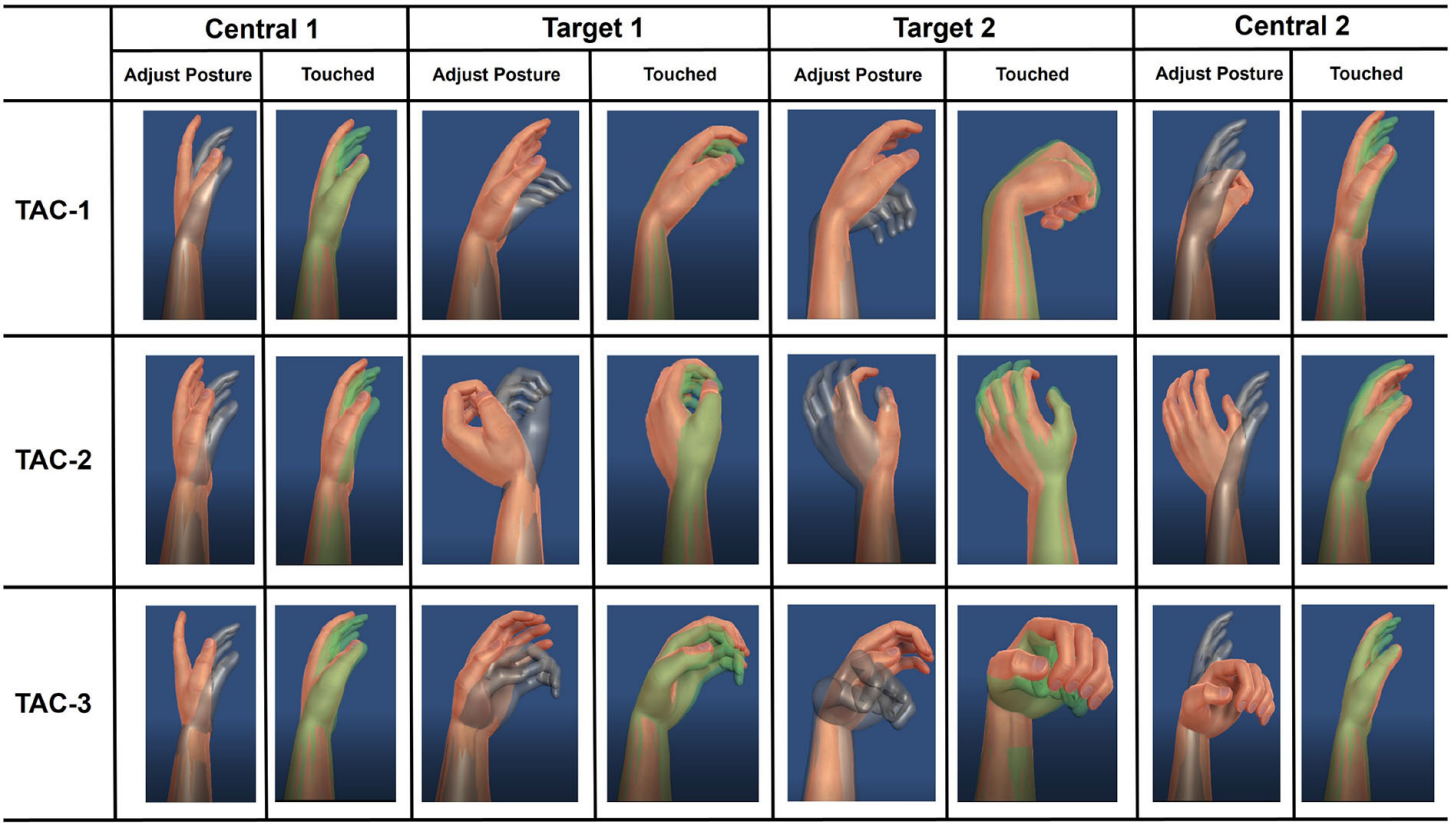
Illustration of the target achievement control (TAC) test designed in this paper. Participants need to control the virtual hand with only EMG electrode sleeve to complete the posture shown as target (gray hand), if the controlled hand is recognized as touching the target hand, it will turn green. When the green color lasts for 0.5 s dwell time, the posture is completed and the target hand changes to the next posture. For each TAC test, participants were required to achieve different target postures (Target 1 and Target 2), and the starting and ending with the central position (Central 1 and Central 2). Each participant was required to complete TAC-1, TAC-2, and TAC-3 test. For detailed TAC test parameters and the 3-DOFs target joint angles for each group of TAC tests, please refer to the Table 2.

In this study, four participants (Sub.3, Sub.4, Sub.5, and Sub.8) were invited to conduct the experiment. The illustration of the TAC test experiment can be found in Figure 4. Participants needed to control the virtual hand with only sEMG electrodes sleeve to touch the target posture. When the target is achieved within acceptable target tolerance, the virtual hand turns green. If the virtual hand achieves the target and stays for the pre-set dwell time, the target posture is completed and target changes to next posture. In order to challenge the difficult task, for each target posture, the angle was changed in all 3-DOFs simultaneously, we set the same acceptance tolerance to ±5°, which was the same as Simon et al. (2011). Considering that real-time motion regression is more difficult than motion classification, we reduced the dwell time to 0.5 s and shortened the time limitation of each trail to 30 s accordingly. We designed three trials TAC tests: TAC-1, TAC-2, and TAC-3. For each trial, the target posture started from the central position (denote as Central 1 in Figure 4) and ended with the central position (denote as Central 2 in Figure 4) after all postures were completed in this trial. We summarize the important TAC parameters and the angular information of the 3-DOFs for each target posture in each TAC-1, TAC-2, and TAC-3, respectively, please refer to Table 2.

**Table 2:** TAC test parameters and target motion details.

**Parameters**		**Setting**
DOF number		3
Dwell time		0.5 s
Target tolerance		±5°
Trial time limitation		30 s
**TAC test**	**Posture**	**Joint angles**
TAC-1	Central 1	DOF1: 0°; DOF2: 0°; DOF3: 0°
	Target 1	DOF1: 15°; DOF2: 15°; DOF3: 15°
	Target 2	DOF1: 30°; DOF2: 30°; DOF3: 30°
	Central 2	DOF1: 0°; DOF2: 0°; DOF3: 0°
TAC-2	Central 1	DOF1: 0°; DOF2: 0°; DOF3: 0°
	Target 1	DOF1: -15°; DOF2: 15°; DOF3: 15°
	Target 2	DOF1: -30°; DOF2: 30°; DOF3: 30°
	Central 2	DOF1: 0°; DOF2: 0°; DOF3: 0°
TAC-3	Central 1	DOF1: 0°; DOF2: 0°; DOF3: 0°
	Target 1	DOF1: 15°; DOF2: -15°; DOF3: 15°
	Target 2	DOF1: 30°; DOF2: -30°; DOF3: 30°
	Central 2	DOF1: 0°; DOF2: 0°; DOF3: 0°

The actual motion performance of each posture for each trial can be checked in Figure 4.

We used completion rate curve to evaluate the TAC results. Within the 30 s time limit, we calculated the completion rate at each time point by obtaining the number of completed postures, and plotted as a completion rate curve:


(10) $$\begin{array}{l} CR\left(t\right)=\frac{N_{completed}\left(t\right)}{N_{postures}} \end{array}$$

Where *CR* means completion rate, *t* is the time point. *CR* will be calculated from the trial beginning until time limit. *N*_*completed*_(*t*) is the completed postures number at *t*. For all TAC-1, TAC-2 and TAC-3. According to Figure 4 and Table 2 the posture numbers *N*_*postures*_ = 4.

## 3. Results

### 3.1. Joint angles estimation

According to Figure 2, in the Task 1 of ***Exp.3***, we obtained four experimental data: (1) Raw sEMG signal; (2) Estimated joint angles by the CW-CNN regression model; (3) System joint angles output (after Adaptive Kalman Filter); (4) Measured joint angles from motion capture system. We calculated the CC between the system joint angles output and the measured angles for each of the 3-DOFs, respectively. And we analyzed CC not only according to different DOFs, but also according to different movement types (single, double-mixture, and triple mixture).

**Figure 5:**
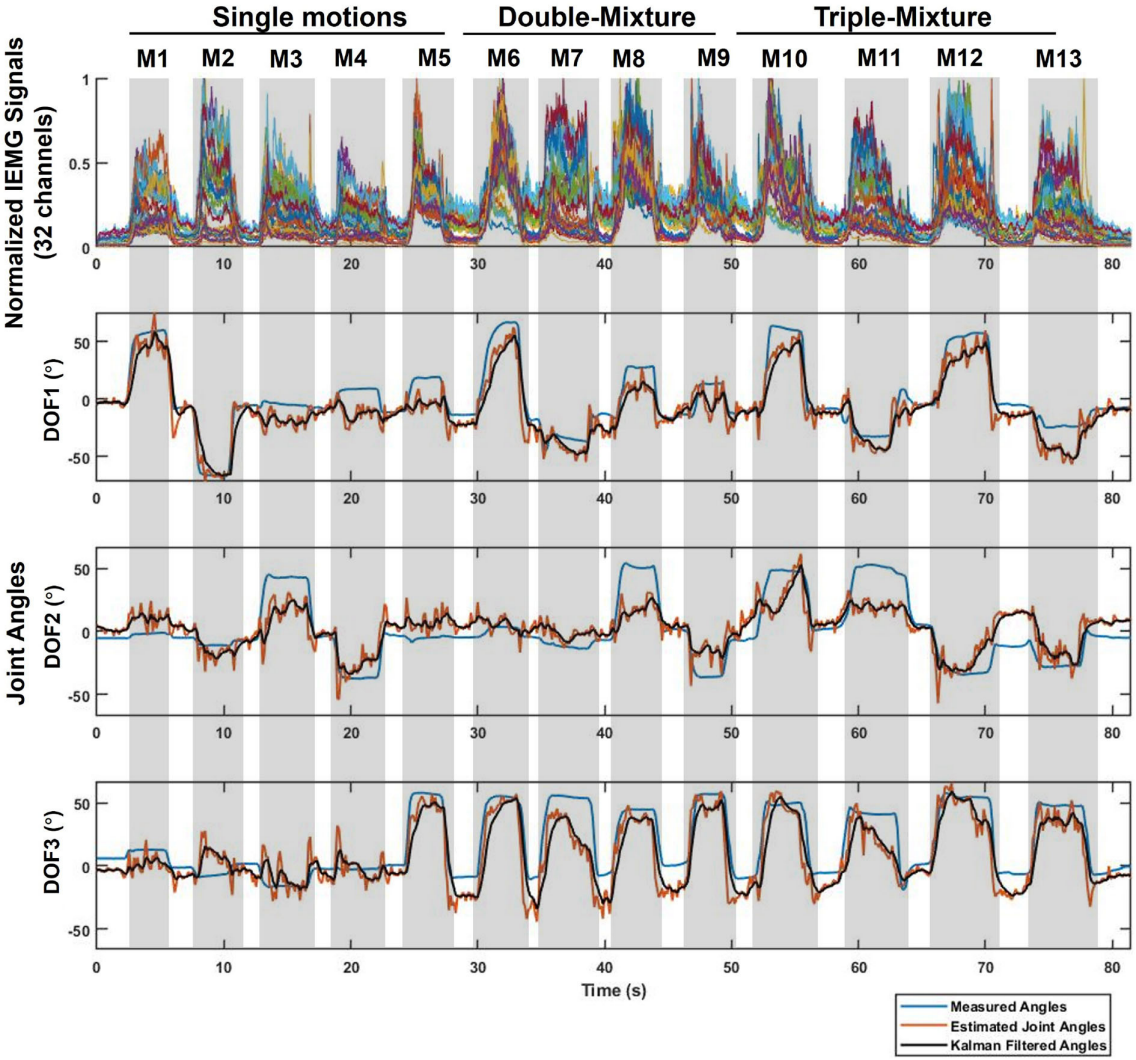
Example real-time experimental results in Task 1 for 3-DOFs joint angles (Sub.1, left hand). The evaluated movements (M1-M13) correspond to the normalized IEMG signals for each 32 channels and the 3-DOFs joint angles. For joint angels: Blue line represents the measured data acquired from the Perception Neuron Motion Capture System; Red line is the estimated output from CW-CNN regression model; Black line denotes the smoothed output *via* Adaptive Kalman Filter. The prediction CC between black line and blue line of each DOF is 0.9406 (DOF1), 0.8030 (DOF2), and 0.9016 (DOF3) respectively.

**Figure 6:**
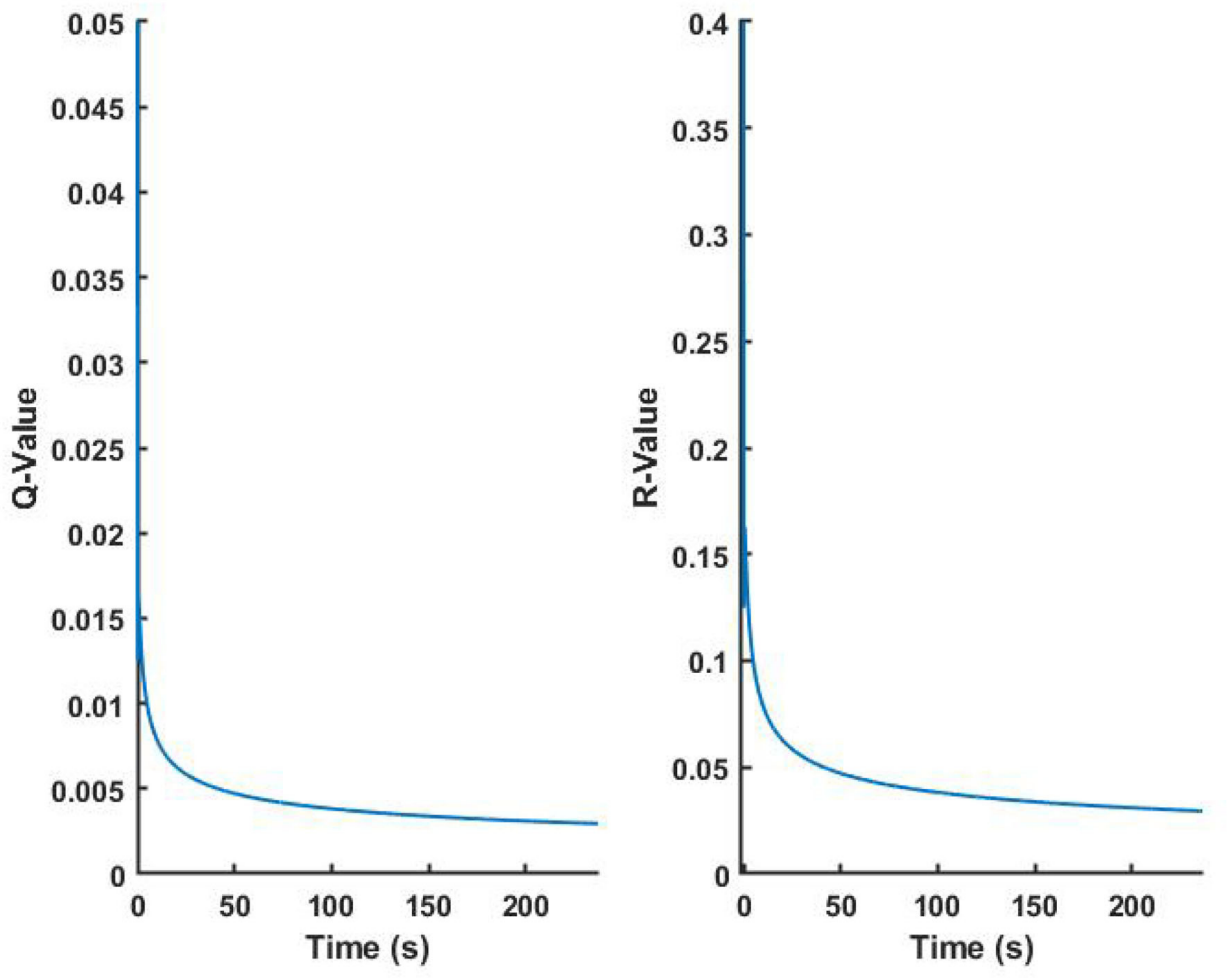
Adaptive results of Kalman hyperparameters *Q* and *R* in real-time experiments.

After we obtained the data through LSL, we corresponded the data by time using LSL timestamp, and converted the raw sEMG signal into normalized IEMG signal according to the MVC of the real-time experiment for judging motions. The real-time estimation result of Sub.1 is shown in Figure 5, CC value of the 3-DOFs joint angles were 0.9406, 0.8030, and 0.9016, respectively. The top figure shows the 32 channels normalized IEMG signals, since the participants were asked to complete the specified motions in order, the hand motions could be corresponded to the order of M1–M13 according to the IEMG. For joint angles curves, blue line indicates the measured true angles, red line indicates the estimated angles using CW-CNN regression model. We can see oscillations from the direct output (red line), and if it is sent directly to the prosthetic hand, there is a risk that the motors will be damaged due to the sudden oscillations. Adaptive Kalman Filter was applied to smooth the angular output of the real-time estimation (black line in Figure 5). All Kalman parameters were updated in the real-time iterations. We set the initial values of the *Q* and *R* to 0.05 and 0.4, respectively, and according to Equation (7) and (8), we were able to observe the asymptomatic convergence of the two hyperparameters (Figure 6).

Since our goal in this paper is to propose a real-time control system, in this paper we only compared the CC between the system output (Figure 5, black line) and the measured angles (Figure 5, blue line) to evaluate the prediction accuracy of the real-time control system.

**Figure 7:**
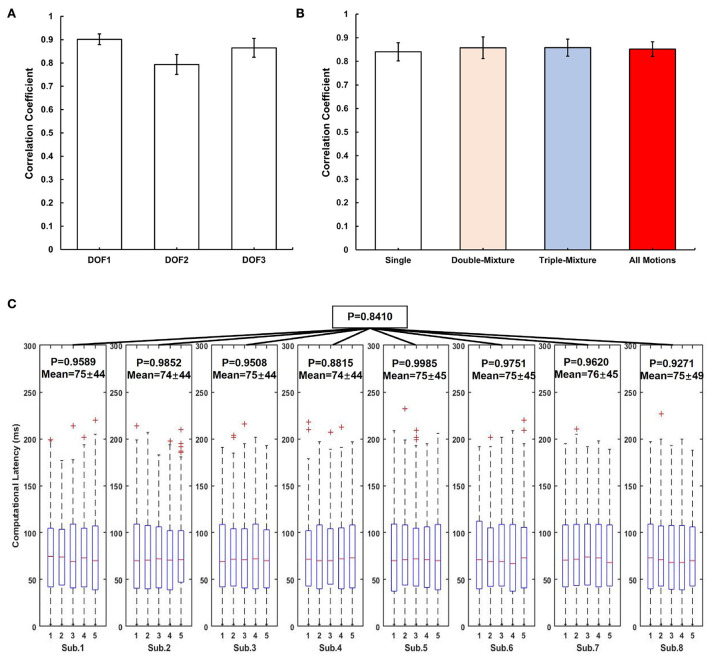
Real-time experimental results of all participants for system evaluation. **(A)** Average CC results over the 3-DOFs joint of participants, error bar indicates the standard error. **(B)** Average CC results for different types of motions, the error bar indicates the standard error. **(C)** Computational latency analysis result using ANOVA. The red mark of the box plot denotes the outliers of the data.

Figure 7A shows the average CC results over the 3-DOFs joint of the eight participants. The CC of DOF1 was 0.9016 ± 0.023, DOF2 was 0.7936 ± 0.0427, and DOF3 was 0.8650 ± 0.0401. We compared the average CC results for all participants for single motions, double-mixture motions, triple-mixture motions and all motions by segmenting the data, as shown in Figure 7B. The average CC result for single motions was 0.8404 ± 0.039, for double-mixture motions was 0.8574 ± 0.046, and for triple-mixture motions was 0.8580 ± 0.036, average CC of all motions for all participants was 0.8519 ± 0.036. The results show that the proposed system is also suitable for estimating not only single motions, but also mixture motions in real time.

### 3.2. System computational latency analysis

The process of converting the raw sEMG signal to IEMG signal and calculating the IEMG signal to joint angle by CW-CNN model in the real-time control system consumes computational time, which can cause some latency in the real-time process. In order to analyze the latency generated by the proposed system in real-time control, we collected the time consumption series during ***Exp.3***.

In this paper, we obtained 5 min of computational latency data from participants using LSL during real-time experiment. We used one-way ANOVA to prove the stability of the system latency, and set significance level to 0.5 to indicate that there is no significant variability between the computational latencies. We analyzed the computational latency of the system in two steps: (1) For each participant, we divided the latency data into five groups, each group data indicates the system latency data in each minute, then used ANOVA to calculate the *p*-value of the five groups latency data; (2) For all participants, we calculated the *p*-values of the eight participants' computational latencies *via* ANOVA. Figure 7C shows the analysis result of computational latency.

### 3.3. Virtual hand control

In this paper, we used Unity engine to build a virtual hand motion program that can perform the 3-DOF movements for a demonstration of real-time control. The virtual hand can receive the joint angles by recognizing the system outputs *via* LSL in real-time. In order to record separate videos of the left- and right-handed demonstrations, we programmed both hands for control.

### 3.3.1. Real-time control demonstration results

Participants were asked to complete the 13 specified motions (M1-M13). Several demonstration videos are available in the Supplementary material, please refer to Supplementary Movie S2 (Sub.8, right hand), Supplementary Movie S3 (Sub.4, left hand) and Supplementary Movie S4 (Sub.4, left hand; motion order changed).

### 3.3.2. TAC test results

**Figure 8:**
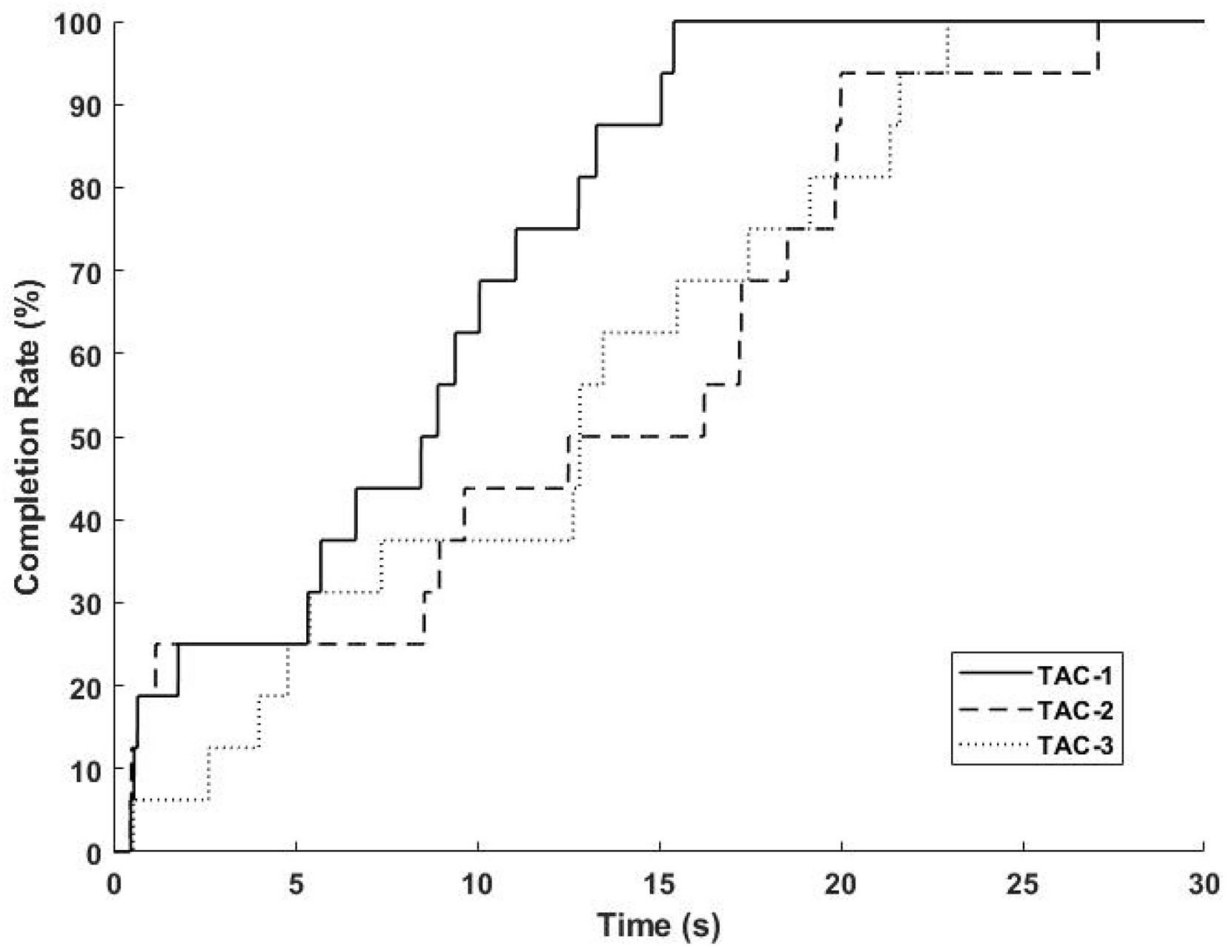
Average completion rate curves for all TAC trials. Solid line indicates the performance during TAC-1; Dashed line indicates the performance during TAC-2; Dotted line indicates the performance during TAC-3.

**Figure 9:**
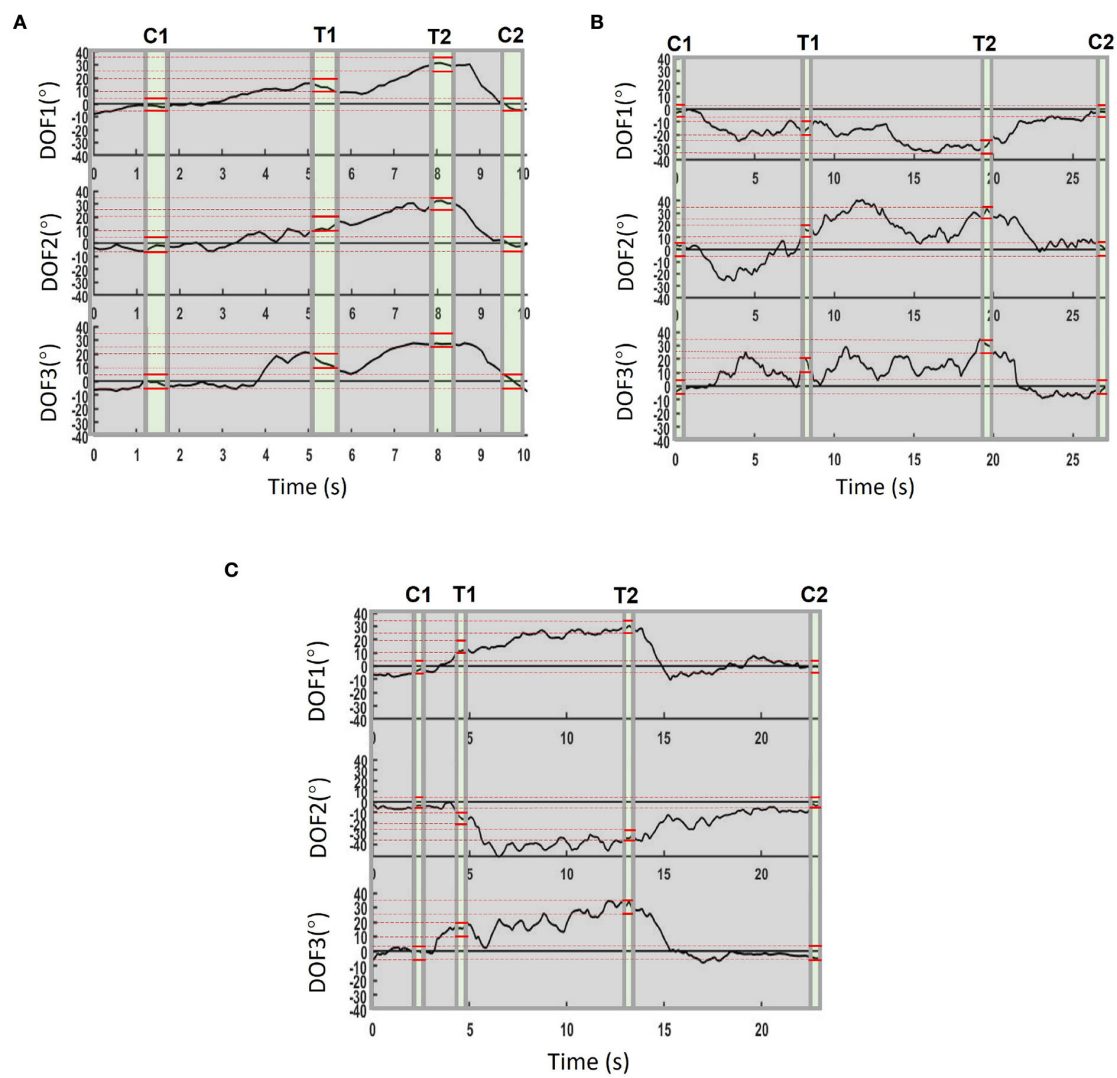
Example real-time regression trajectory (Sub.4). C1 and C2 mean Central 1 and Central 2 posture, T1 and T2 mean the Target 1 and Target 2. The 0.5s dwell duration is represented as green color, the gray areas denote the movement adjustment duration, the red lines indicate the upper and lower boundaries of the acceptance tolerance range of the target posture. **(A)** Trajectory in TAC-1, trial ended at 9.9 s in this result. **(B)** Trajectory in TAC-2, trial ended at 27.1 s in this result. **(C)** Trajectory in TAC-3, trial ended at 22.9 s in this result.

Demonstration videos of TAC test experiment are provided, please refer to Supplementary Movies S5–S7. During TAC test, all participants completed the trials for all TAC-1, TAC-2, and TAC-3 within the time limit (30s, Table 2). Figure 8 is the average completion rate curves result, solid line indicates the performance during TAC1, dashed line indicates the performance during TAC-2, and dotted line indicates the performance during TAC-3. Moreover, we show the trajectory plot of all TAC trials of the 3-DOFs for one of the participants (Sub.4) as Figures 9A–C, the green area denotes the dwell time duration, gray area denotes the adjustment duration, and red lines are the upper and lower boundaries of the acceptance tolerance (±5°, Table 2) range of target posture. We will discuss the regression effect based on these results in Section 4.4.

## 4. Discussion

We proposed a real-time control system for virtual hand control. We analyzed regression accuracy by calculating Pearson CC between system angles output and measured joint angles. We also analyzed the computational latency of the system by collecting 5 min of latency data during real-time experiment of each participant. Furthermore, participants controlled a virtual hand using their sEMG signals to complete the 13 daily motions and TAC test. In this paper, we proposed a real-time control system, the good performances (accuracy and computational latency) of the system are the main contributions of this paper. When using the virtual hand as demonstration object, we can focus on analyzing the proposed system. If we control a real prosthetic hand, in addition to the computational latency, the real-time performance will also relate to the data transmission speed, hardware delay, etc., which should be other topics.

### 4.1. Real-time data processing

The IEMG signal was less noisy compared to the raw sEMG signal and the signal due to muscle activity becomes more pronounced after the normalization process based on the MVC. Under real-time conditions, the sliding window was always slid to the latest acquired raw sEMG signal to prevent the delay caused by the signal transmission. One of the examples of real-time sEMG processing results can be found in Supplementary Figure S2.

### 4.2. Real-time regression accuracy and effect of adaptive Kalman filter

The real-time joint angles estimation results in Figure 5 show that there are oscillations in the estimated joint angles output (red line). The reason could be caused by the low sampling rate of the estimated joint angles (about 7.5 Hz), which could be caused by the calculation latency and the special handling of the sliding window. The computational latency was unavoidable, however, each sliding window took the latest batch of sEMG signals for processing, and the system processed them until the next data acquisition, while new sEMG signals were still being generated continuously during the sliding window processing, so there was a certain amount of signal loss during the processing procedure from the previous processing to the next processing. We think this should be the main reason for the oscillations. Despite the oscillations occurred in the estimated output, we could still see the expected estimated motions from the curve, thus, we only need to smooth the output angle of the model so that we can use the joint angles directly for safe real-time control of the prosthetic hand. The Adaptive Kalman Filter was applied to smooth the estimated joint angles. Before using the Adaptive Kalman Filter, we tried to use a conventional Kalman Filter to handle the output oscillations, we could only adjust the hyperparameters *Q* and *R* manually. After many attempts, we found that setting to *Q* = 0.05 and *R* = 0.4, the Kalman Filter performed our expected results. However, this still does not indicate that the artificially pre-set hyperparameters are the optimal solution for the real-time situation. Therefore, we considered the improvement of the conventional Kalman Filter to a hyperparametric Adaptive Kalman filter. In our expectation, the two hyperparameters should converge to their optimal solutions, respectively as time increases. Finally, we considered an update mechanism using variance estimate-based method (Mehra, 1970) to predict the hyperparameters at the next moment, achieving our expected results (Figure 6). Even though we used the Adaptive Kalman filter, we still needed to pre-set the initial hyperparameters, so we set the initial values of the *Q* and *R* to 0.05 and 0.4, respectively. For all participants, *Q* and *R* converged to near 0.002 and 0.02, respectively.

Figure 7A shows the CC results for different DOFs, we can find that although we offline trained and estimated only single motions (M1–M5) analyzed in Qin et al. (2021), we can still see the expected real-time motions in the system estimation results (Figure 5) even after adding mixture motions (M6–M13). Figure 7B shows the CC results for single motions, double-mixture motions, triple-mixture motions and all motions. We can find that for mixture motions (double-mixture and triple-mixture motions), the estimation average CCs were accurate and nearly at the same level than that of the single motions. Therefore, we can conclude that the proposed CW-CNN based real-time control system can not only regress estimate the 3-DOFs joint angles simultaneously, but also achieve a good performance for mixture motions. This gave us a lot of confidence to conduct the subsequent 3-DOFs TAC test.

### 4.3. Computational latency of the proposed system

We define the duration which consumed by the following process as the computational latency: (1) From raw sEMG signal to normalized IEMG signal, (2) CW-CNN regression model calculation, (3) Using Adaptive Kalman Filter to obtain system output. We used PyQt5 to build the whole system framework, and everything in the *update*() function was looped regularly *via* *QtCore*.*QTimer*(). We used *time*.*time*() in Python3 to not only get the start time before each acquisition of the latest sEMG signal through sliding window, but also get the end time after using Adaptive Kalman Filter, and then the time difference was calculated by subtraction and converted to milliseconds (ms) units. Readers can refer to **Pseudo Code S1** in Section 2. From Figure 7C, firstly, we can focus on the result for each participant during the whole real-time experiment. Let us take Sub.1 as an example, the *p*-value for these 5 min is 0.9589 (>0.5), indicating that the computational latency of the control system is stable at 75 ± 45 ms during the real-time experiment. Similarly, for Sub.2–Sub.8, all of the *p*-values are higher than 0.5, and the average latency is 74–76 ms. Then we can focus on the computational latency for all participants. The *p* = 0.8410 (>0.5) shown that the proposed system maintains a stable computational latency during the experiment even on different days. In the contraction of skeletal muscle, there is a delay between electrical activity and force detection, this delay in electromechanical coupling is about 30–100 ms (Cavanagh and Komi, 1979), and by watching the videos in Supplementary material, the computational latency of about 75 ms essentially restores the process of electrical signals to muscle activity, and is acceptable. In addition, Considering that the sliding window length is 120 ms in duration, and with the 75 ms latency still within the real-time control constraint of about 300 ms (Tam et al., 2020). However, from Figure 7C, by observing the latency box plots for a specific participant, it can be seen that although the latency sequences are stable, latencies still floating within a certain range, i.e., with a standard deviation of ±44 ms. We consider that this was due to the uncontrollable delay generated by this real-time control system using LSL to receive the sEMG signal and continuously send the experimental data at the same time (Figure 2). It might also be caused by the system hardware of the PC used for the experiment.

### 4.4. TAC test and regerssion trajectory emphasis in 3-DOFs

The purpose of designing the TAC test was to verify that users could control the virtual hand using the proposed system to reach the target motions within time limit in real-time, and to emphasize the effect of simultaneous regression of 3-DOFs. The real-time regression effect can be proved in this experiment, and demonstration video of TAC-1, TAC-2, and TAC-3 are provided in Supplementary Movies S5–S7.

Figure 8 indicates how the participants completed the target postures within the trial time limit, we can find that all participants completed all postures. In Figures 9A–C, the dwell phases were shown as green areas based on these dwell duration information. Take TAC-1 as an example, according to Table 2, target angles of the four target postures were 0°, 15°, 30°, and 0°, respectively, for all 3-DOFs, which means, with ±5° acceptance tolerance, the ranges were [–5°, 5°], [10°, 20°], [25°, 35°], and [–5°, 5°], respectively. The same analysis was applied to TAC-1, TAC-2, and TAC-3 for all participants. We used bold red lines to express the upper and lower boundaries of the acceptance tolerance ranges in trajectory curves. According to the above explanation, from trajectory curves, we found that participants tracked the target postures, and joint angles were regression in 3-DOFs in real-time. Compared to Figure 5, in TAC test, the angles stayed for dwell time at different intermediate angles instead of changing from 0° to the maximum angles only, which is the significant proof to distinguish real-time regression from real-time classification. Moreover, we can check highly desirable regression result in 3-DOFs simultaneously. Even though the angles changed within the range during posture dwell time, we consider this was due to slight muscle contraction and relaxation during motion maintenance. To maintain a motion, we always adjust our muscle force to stay, this is the difficulty of real-time regression control compared to motion classification.

## 5. Limitations and future work

We conducted experiments for eight participants and evaluated the proposed real-time control system through analysis of regression accuracy (CC), computation latency and virtual hand control demonstration. In addition, four of the participants joined the TAC experiment to test the real-time regression performance of the proposed real-time control system. Due to some limitations of this study, we proposed the following future work.

### 5.1. Apply the proposal to a practical prosthetic hand control task

In this paper, we discussed the computational latency for real-time joint angles estimation. If we use a real prosthetic hand, besides the latency of system processing, the control latency also depends on the hardware performance of the machine. Moreover, in the future, if we want to control the prosthesis directly, in addition to the computational latency, we also need to consider the latency caused by the command transmission as well as the performance of the prosthesis hardware.

### 5.2. Introduce force and tactile feedback mechanism to real-time control system

In the future, when we use an actual prosthetic hand for real-time control, it will be interesting to include experiments on grasping objects based on high-precision estimation of multi-joint angles. Thus, regression prediction of grip force (Baldacchino et al., 2018; Chen et al., 2020) should also be considered. For enhancing the ability of amputees to interact with objects, tactile feedback is important (Navaraj et al., 2019), integrated biomimetic tactile sensors (Liang et al., 2019; Abd et al., 2020; Romeo et al., 2021) and a feedback actuation mechanism (Stephens-Fripp et al., 2018) should be designed in the future.

### 5.3. Test on actual amputees

In this study, we only tested the real-time control system on healthy participants. However, it is hard to prove the similar level can be achieved for actual amputees or not. The real-time performance of amputees will depend on the level of muscle residuals. We regret that we were unable to invite actual amputees to participate in the real-time experiments. In the future, the task to apply the proposed real-time control system to actual amputees based on the data acquisition methods in this paper should be mentioned.

## 6. Conclusion

In this paper, we proposed a deep learning regression-based real-time control system for virtual hand control based on CW-CNN regression model. The system estimated 13 daily motions (including single motions, double-mixture motions and triple-mixture motions) in real time based on sEMG signal. We used a virtual hand model as a demonstration of real-time control. In real-time experiments, eight participants controlled the virtual hand to complete the specified daily motions, and four of them conducted TAC test. The experimental results show that: (1) The proposed real-time control system has the good prediction effect for not only single motions but also mixture motions; (2) The system has a stable (*p* > 0.5) computational latency (average 74–75 ms), and this latency is acceptable in real-time control; (3) The proposed control system can complete the specified TAC test, providing more proof of motion regression accuracy in real-time processes. The usability of this control system has been verified by a demonstration of controlling a virtual hand.

## Data availability statement

The raw data supporting the conclusions of this article will be made available by the authors, without undue reservation.

## Ethics statement

The studies involving human participants were reviewed and approved by Tokyo Institute of Technology. The patients/participants provided their written informed consent to participate in this study.

## Author contributions

ZQ designed and operated the experiments, developed experiment programs, Adaptive Kalman Filter program and the source code of real-time control tool GUI, adjusted the program of virtual hand, analyzed the results, and wrote and edited the manuscript. ZH operated the experiments and analyzed the results. YL developed the Kalman Filter of real-time control system and did statistical analysis. SS developed virtual hand program. YK directed the study and guided the manuscript. All authors contributed to the article and approved the submitted version.
