# Supplementary material for: A CW-CNN regression model-based real-time system for virtual hand control
Source: Front Neurorobot. 2022 Dec 21;16:1072365. doi: 10.3389/fnbot.2022.1072365 (PMC9812573; doi:10.3389/fnbot.2022.1072365)
Supplement: Supplementary file 8 [file Data_Sheet_1.PDF]

Supplementary Material

1 SUPPLEMENTARY DATA

Figure S1: Data Acquisition Devices.

Figure S2: Real-time processing results of sEMG signals in the Data Processing Module.

Table S1: CW-CNN regression model training detail.

Table S2: Participants information.

Pseudo Code S1: Calculation routine for calculating latency.

Movie S1: Usage of proposed real-time control tool GUI.

Movie S2: Virtual hand real-time control demonstration (Sub.8, right hand).

Movie S3: Virtual hand real-time control demonstration (Sub.4, left hand).

Movie S4: Virtual hand real-time control demonstration (Sub.4, left hand; Order changed).

Movie S5: TAC-1 Experiment (Sub.3).

Movie S6: TAC-2 Experiment (Sub.3).

Movie S7: TAC-3 Experiment (Sub.3).

2 SUPPLEMENTARY TABLES AND FIGURES

2.1 Figures

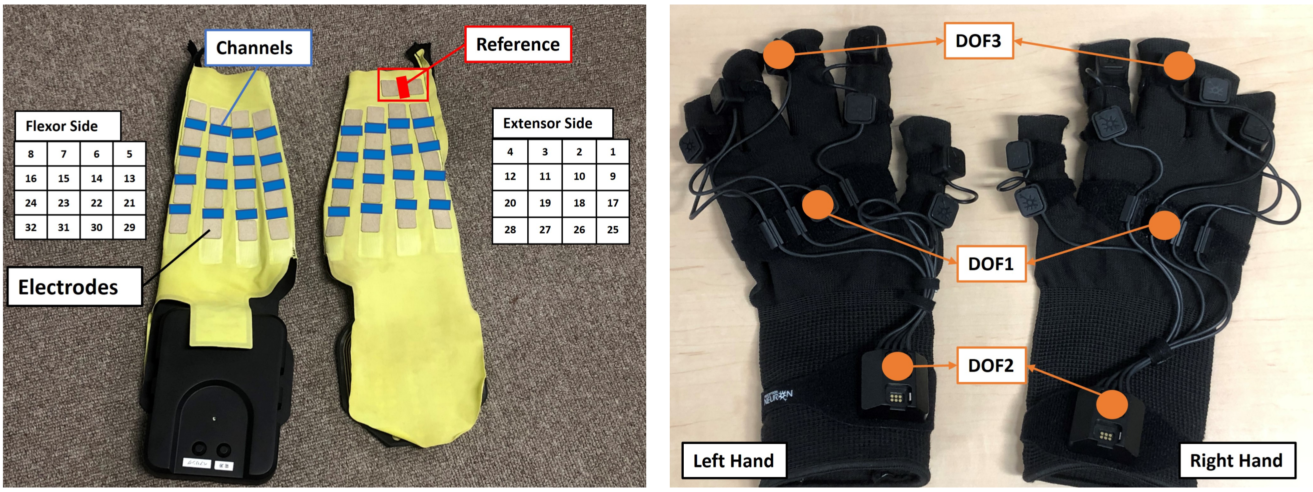

Figure S1: Data Acquisition Devices. **(A)** Bipolar multi-array electrodes (32 channels, the picture shows the right hand) for sEMG signal acquisition. The red box indicates that the two electrodes are shared as reference, the blue part indicates the channel that is shared by the upper and lower electrodes. The channel matrixes on both flexor and extensor side are given, the number denotes the channel number. sEMG signal was sent to PC through the black box via Bluetooth. **(B)** Perception Neuron Motion Capture System glove (both hands) for joint angles acquisition. We use the three makers which shown as orange circles to obtain the angle data in each of the 3-DOFs.

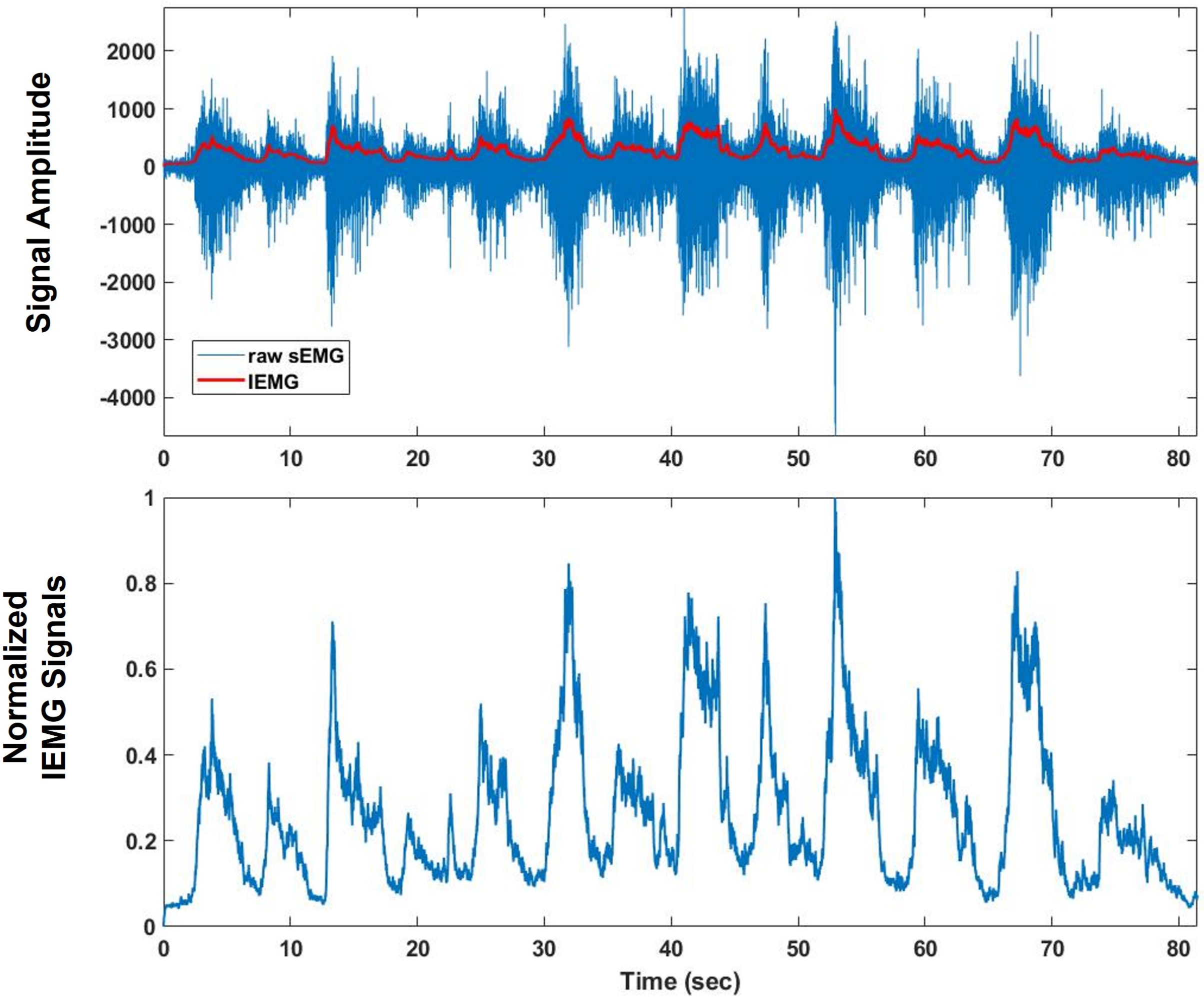

Figure S2: Real-time processing results of sEMG signals in the Data Processing Module (Sub.1, ch5). The top figure shows the process from the raw signal to the IEMG, the blue line represents the raw signal, and the bold red line is the IEMG signal. The bottom figure shows the results of the IEMG normalization process based on the MVC maximum and minimum values for this channel.

2.2 Tables

Table S1. CW-CNN regression model training detail. The training procedure using PyTorch 1.3.1, GeForce RTX 2080 GPU and CUDA10.1. The convolutional layer is denoted as *Conv layer* and the fully connected layer is *FC layer*. *LR* means learning rate, and *CV* is abbreviation of cross-validation.

**Exp.**	**Training layer**	**LR**	**Validation**	**Epochs**	**Duration**
Exp.1	*Conv.* layer<br>FC layer	0.005	10-fold CV	10	≈30 min
Exp.2	FC layer	0.002	5-fold CV	5	≈2.5 min

Table S2. Participants information. There are Eight participants, four male and one female, aged from 21–26. Due to the limited size of the multi-array electrode sleeves, the choice of the right or left hand depends on whether the forearm size of the participant fits better in the left- or right-hand sleeve.

ID	Gender	Age	Handedness
Sub.1	M	22	Left
Sub.2	M	25	Right
Sub.3	F	25	Left
Sub.4	M	28	Left
Sub.5	M	26	Left
Sub.6	M	21	Left
Sub.7	M	23	Right
Sub.8	M	24	Right

2.3 Pseudo Code

Algorithm 1 Calculation routine for calculation latency

function $CalculationLatency(\,)$
    $time_{start} \leftarrow GetCurrentTime()$
    $EMG_{raw} \leftarrow GetEMG()$
    $IEMG \leftarrow Filter(abs(EMG_{raw}))$
    $IEMG_{norm} \leftarrow Normalization(IEMG)$
    $Angles \leftarrow CWCNN(IEMG_{norm})$
    $Output_{system} \leftarrow AdaptiveKalmanFilter(Angles)$
    $time_{end} \leftarrow GetCurrentTime()$
    return $time_{end} - time_{start}$
end function
